# Regulatory mechanisms triggered by enzyme interactions with lipid membrane surfaces

**DOI:** 10.3389/fmolb.2023.1306483

**Published:** 2023-11-30

**Authors:** Jie Yu, David D. Boehr

**Affiliations:** Department of Chemistry, The Pennsylvania State University, University Park, PA, United States

**Keywords:** protein-membrane interactions, allosteric regulation, conformational change, signaling, lipid metabolism, lipid kinase, phosphatidylinositol phosphate lipids

## Abstract

Recruitment of enzymes to intracellular membranes often modulates their catalytic activity, which can be important in cell signaling and membrane trafficking. Thus, re-localization is not only important for these enzymes to gain access to their substrates, but membrane interactions often allosterically regulate enzyme function by inducing conformational changes across different time and amplitude scales. Recent structural, biophysical and computational studies have revealed how key enzymes interact with lipid membrane surfaces, and how this membrane binding regulates protein structure and function. This review summarizes the recent progress in understanding regulatory mechanisms involved in enzyme-membrane interactions.

## 1 Introduction

Protein-membrane interactions play a crucial role in cell signaling and membrane trafficking (Cho and Stahelin, 2005; Niggli, 2005; Leonard and Hurley, 2011). Membranes serve as sites for signaling activity and as dynamic scaffolds for the recruitment of signaling molecules. These interactions can re-localize enzymes to substrates on membranes and regulate enzyme function through allosterically-induced conformational changes. Allosteric regulation upon membrane binding allows the enzyme to respond to changes in membrane lipid composition and allows for different activities in different subcellular compartments. Allosteric sites may also serve as targets for small molecule inhibitors for drug development, which may act to block appropriate protein-membrane interactions and/or conformational changes necessary for protein function. In this Review, we focus on peripheral membrane binding enzymes that leverage various strategies for reversible membrane interaction, often leading to changes in enzyme function. Two major methods to facilitate membrane interactions are modular membrane-targeting domains that recognize specific lipids in the membrane (Lemmon, 2008; Stahelin, 2009) and amphipathic secondary structures (Drin and Antonny, 2010; Madsen et al., 2010). There are also post-translational modifications (e.g., palmitoylation) that add a hydrophobic anchor that can act together with protein components to relocalize proteins to membranes and regulate enzyme function.

Here, we emphasize recent biophysical studies that help to describe the mechanisms of allosteric regulation upon enzyme-membrane interactions, help to identify common themes amongst these proteins, and provide a future road map for further understanding this critical class of enzymes. We divide these enzymes into two major groups, those that primarily use an amphipathic helix for membrane engagement and those that use specialized membrane-interacting domains, including C1/C2, PH, PX and FERM domains. The examples are meant to be illustrative, not exhaustive.

## 2 Amphipathic helix-containing enzymes

Many membrane-associated proteins contain an amphipathic α-helix that is often unfolded in solution but becomes folded upon membrane interaction. Such a protein segment may initially be attracted to the membrane through electrostatic interaction, but then folds into an α-helix with its nonpolar residues inserted into the membrane while polar residues face the lipid head groups. It has been proposed that these amphipathic helices especially bind to membranes with high curvature owing to their lipid packing defects (Drin and Antonny, 2010).

### 2.1 Phosphocholine cytidylyltransferase

**FIGURE 1:**
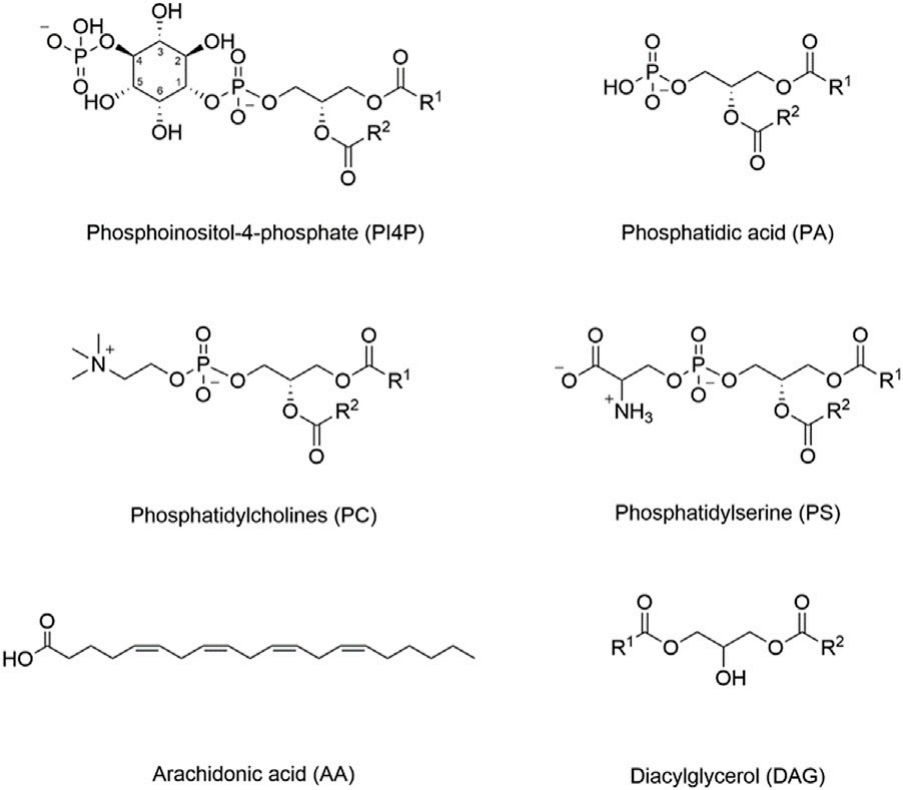
Structures of important membrane lipids. The inositol ring is numbered in phosphoinositol-4-phosphate (PI4P), as different hydroxyl groups can be phosphorylated to generate different phosphoinositide lipids (PIPs). PIPs can also be phosphorylated at more than one position. For example, PI(4,5)P_2_ represents phosphorylation at both the 4- and 5-positions. Different internal membranes are enriched in different PIP lipids, and some PIP-interacting domains may have increased affinity for PIPs phosphorylated at specific positions.

Two well-studied enzymes known to interact with membranes through their amphipathic helices are phosphocholine cytidylyltransferase (CCT) and phospholipase A_2_ (PLA_2_). CCT is a rate-limiting and regulatory enzyme of phosphatidylcholine (PC) metabolism (Vance and Choy, 1979). Chemical structures of PC and other important lipids are illustrated in Figure 1. CCTα/CCT1 and CCTβ/CCT2 are two major isoforms in eukaryotic organisms, which have similar structures (Lykidis et al., 1999) and are regulated similarly by membrane lipids. CCT undergoes allosteric activation when binding to membranes, which turns the soluble form (CCTsol) to a membrane-bound form (CCTmem) and boosts the catalytic efficiency by over 200-fold. The work by the Cornell lab (Cornell, 2016; Cornell, 2020) has especially been instrumental in our understanding of how the CCT-membrane binding activates CCT.

**FIGURE 2:**
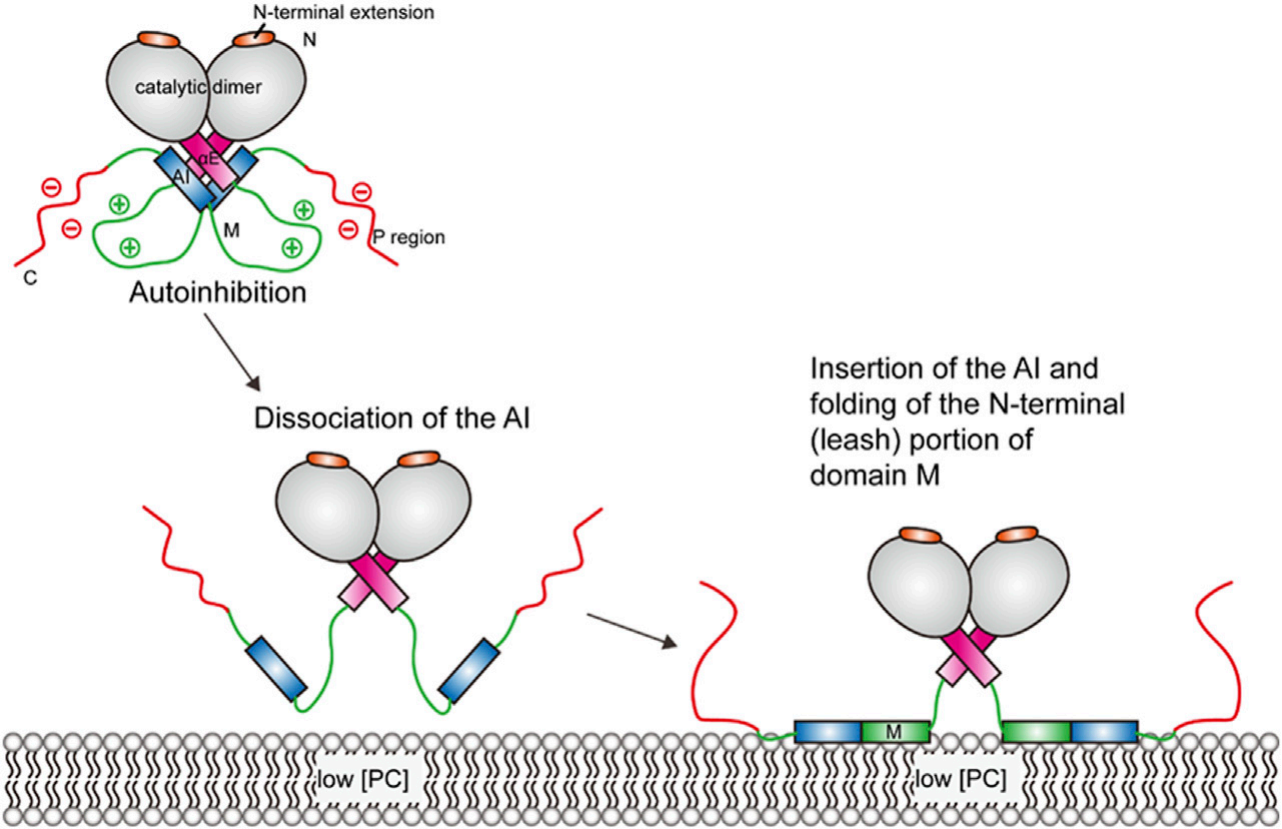
Schematic illustration of CCT activation. The CCT enzyme is kept in an autoinhibited state until interactions with the membrane lead to a series of conformational changes, which enables the amphipathic M domain to interact with the membrane.

Upon membrane binding, the autoinhibitory (AI) segment of the membrane–lipid sensor domain (domain M) dissociates from the four-helix complex (2 αE + 2 AI helices) at the base of the catalytic domain (see Figure 2), as suggested by photo-crosslinking and deuterium exchange analysis (Huang et al., 2013). Molecular dynamics (MD) simulations suggest that this dissociation destabilizes the αE helices and enables it to sample new conformations (Ramezanpour et al., 2018). Following the change in the αE helices, membrane insertion and folding of the “leash” of domain M leads to a conformational change in the linker region (Taneva et al., 2019). The allosteric linker, composed of the αE hinge, the αEC, and the J segment, can mediate the communication between domain M and the active site. Fluorescence resonance energy transfer (FRET) experiments suggest that folding of the allosteric linker can pull the active site close to the membrane (Knowles et al., 2019).

Membrane lipid composition and the phosphorylation status of the C-terminal region are two regulators of CCT membrane binding (Cornell, 2016). High negative charge density and lipid packing defects on the membrane caused by low PC content trigger the membrane binding and the conversion of CCT, leading to PC synthesis. In the absence of high negative charge density and lipid packing defects, the rate of PC synthesis is lower. This feedback regulation helps to maintain cellular PC homeostasis (Cornell, 2016).

Perhaps the most interesting aspect is the dual function of the amphipathic helix domain M: it acts as both an autoinhibitory device in CCTsol and an activating device in CCTmem. Membrane binding of domain M disrupts its autoinhibitory contact with the active site. The deletion of domain M or specifically the AI segment only leads to partial activation. For full activation, domain M must be present (Ding et al., 2012). Amphipathic helices contribute to protein–membrane binding since they can overcome the water–lipid energy barrier (Drin and Antonny, 2010). The amphipathic helix domain M can essentially sense the features of low PC content membrane, enabling membrane binding (Cornell, 2016). Mechanistic studies suggest that the amphipathic helix binds to negatively charged membranes through electrostatic interactions and the hydrophobic effect. With no electrostatic effect, it then relies on the insertion of its hydrophobic residues into lipid packing defects (Drin and Antonny, 2010). This mechanism may be applicable to other membrane binding proteins with similar amphipathic helix. Although there is strong evidence showing that the linker is important for allosteric control, a high-resolution CCT-membrane binding structure in its active form is still not solved (Cornell, 2020), which would provide much needed insight.

### 2.2 Phospholipase A_2_

PLA_2_ catalyzes the hydrolysis of acyl chains at the *sn*-2 position of membrane phospholipids to produce fatty acids that are important for downstream signaling. The activity of PLA_2_ is higher in the presence of lipid aggregates (vesicles, micelles, etc.) than monomeric substrates (Singer et al., 2002; Gelb et al., 2003). The PLA_2_ superfamily consists of 16 groups and many subgroups, and can be described as six types. Each type has a distinctive structure with an active site binds to specific phospholipid substrates and an interfacial surface that can bind to the membranes (Mouchlis et al., 2015; Mouchlis et al., 2018).

The Dennis lab (Dennis, 2022; Mouchlis and Dennis, 2022) has made many important insights into the allosteric interaction between lipid bilayer membranes and PLA_2_, especially through their judicial use of hydrogen–deuterium exchange mass spectrometry (HDX-MS) combined with MD simulations to understand the association mechanism of the recombinant human form of group IVA cytosolic (cPLA_2_), group V secreted (sPLA_2_), group VIA calcium-independent (iPLA_2_), and group VIIA lipoprotein-associated phospholipase A_2_ (GVIIA Lp-PLA_2_); they propose that PLA_2_ exists in at least three forms: the “closed” form in the cytosol, the “open” membrane-associated “unbound” form, and the “open” membrane-associated “bound” form when binding to a substrate (Mouchlis and Dennis, 2022).

HDX-MS studies on the iPLA_2_ with phospholipid vesicles and MD simulations suggest that an amphipathic helix (residues 708–730) close to the active site penetrates the membrane. The hydrophilic residues of the amphipathic helix interact with the headgroups of the lipids and the hydrophobic residues interacts with the fatty acid chains (Hsu et al., 2009; Mouchlis et al., 2015). Studies on sPLA_2_ also show an amphipathic helix working in a similar way (Burke et al., 2008a). HDX-MS indicates that an amphipathic loop (residues 640–648) and the amphipathic helix (residues 708–730) are important for regulating the volume of the binding pocket of iPLA_2_. Indeed, MD simulation studies suggest that the binding pocket volume of iPLA_2_ is larger in the presence of a membrane.

cPLA_2_ contains a protein kinase C (PKC) conserved region 2 (C2) domain and a catalytic domain. HDX-MS showed lower deuteration levels in some peptide regions derived from the C2 and catalytic domains in the presence of phospholipid vesicles (Burke et al., 2008b). According to their model, part of the C2 domain (residues 35–39 and 96–98) penetrates the membrane and “pulls” the catalytic domain to the membrane. Two amphipathic helixes of the catalytic domain (residues 268–279 and 466–470) also interact with the membrane (Burke et al., 2008b; Mouchlis et al., 2015).

Based on the HDX-MS data, two amphipathic helices of Lp-PLA_2_ (residues 114–120 and 360–368) are involved in membrane binding. MD simulations show that the volume of the active site is increased upon membrane binding and the amphipathic helical region (residues 100–130) is responsible for this conformational change (Mouchlis et al., 2022). A site-directed tryptophan fluorescence experiment also indicated that this peptide region is in a more polar environment because of the conformational change in the presence of vesicles.

Altogether, these data support the hypothesis that the membrane binding sites of the PLA_2_s serve as allosteric sites, and that membrane binding changes the enzyme conformation from a closed, inactive form to an open, active form on the membrane surface.

## 3 C1 domain-containing enzymes

**FIGURE 3:**
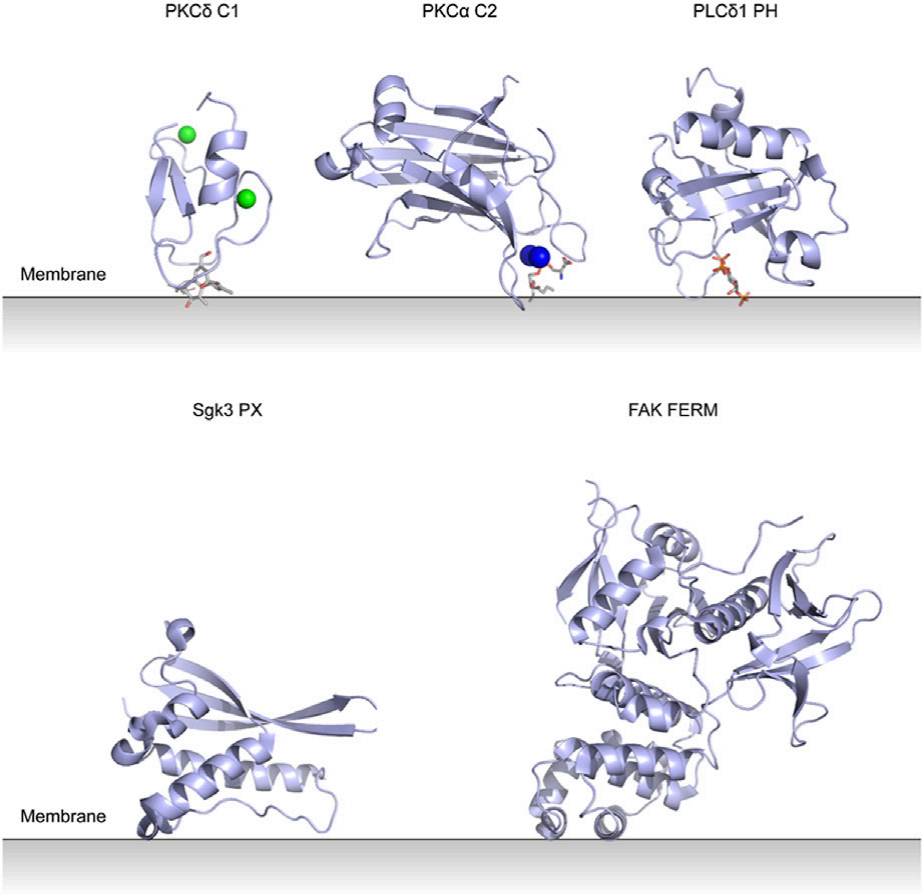
Lipid binding domains on the membrane with their putative membrane-binding pose. PKCδ C1 domain with Zn^2+^ and phorbol-1,3-acetate (PDB entry 1PTR). PKCα C2 domain with Ca^2+^ and phosphatidylserine (PDB entry 1DSY). PLCδ1 PH domain with inositol trisphosphate (PDB entry 1MAI). Sgk3 PX domain (PDB entry 6EDX). FAK FERM domain (PDB entry 2AL6).

PKC conserved region 1 (C1) domains were first identified in PKCs and most of them bind to diacylglycerol (DAG) and phorbol ester (Colón-González and Kazanietz, 2006). They are zinc finger-like domains composed of ∼50 amino acids. The interaction between C1 and DAG is the key part of PKC activation. Structures of PKC membrane binding domains are shown in Figure 3.

### 3.1 Protein kinase C

PKC is a Ser/Thr kinase and is one of the best-studied paradigms of enzymes that are re-localized to membranes and then activated by lipids. All PKC isoenzymes consist of the N-terminal regulatory domain, C-terminal catalytic domain and a hinge between them. Conventional PKC isoenzymes (cPKCs; α, βI/II, and γ) are activated by two second messengers Ca^2+^ and DAG. Novel PKC isoenzymes (nPKCs; ε, δ, θ, and η) are activated by DAG alone. Atypical PKCs (aPKCs; ζ and ι/λ) are regulated by protein-protein interactions.

The activation of cPKCs requires a conformational transition from an inactive cytosolic form to an active membrane -bound form. The inactive form is autoinhibited by trapping the pseudosubstrate (PS) region into the active site of kinase domain. The autoinhibitory interaction is released upon interaction of the regulatory domain with lipid membrane.

**FIGURE 4:**
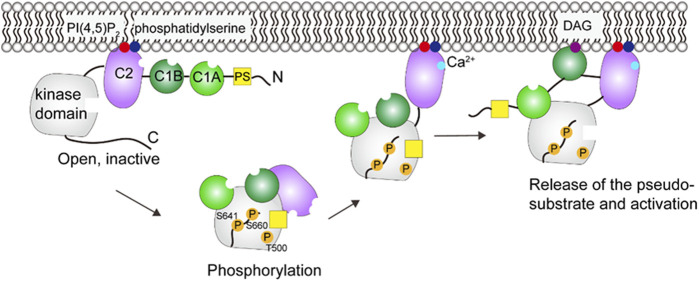
Schematic illustration of PKCβII activation. In the open, inactive form, the C2 domain binds PI(4,5)P_2_ and phosphatidylserine, but the DAG site in C1B is blocked. Phosphorylation and Ca^2+^ binding induces a series of conformational changes that allows the C1B to engage with DAG. These events lead to the activation of PKC. This figure was adapted in part from ref. Antal et al. (2015).

One model (see Figure 4) (Antal et al., 2014) proposes that PKCβII is originally in an open conformation and all the domains are unmasked (Dutil and Newton, 2000). Phosphorylation of PKCβII at three sites (T500, T641, S660) then leads to an autoinhibited conformation (Behn-Krappa and Newton, 1999; Feng et al., 2000; Antal et al., 2015). The Ca^2+^-sensing C2 domain clamps the autoinhibitory PS region in the active site of the kinase domain, and the DAG-sensing C1 domains is masked. Hydrolysis of phosphatidylinositol-4,5-bisphosphate (PI(4,5)P_2_) generates second messengers, DAG and inositol-1,4,5-trisphosphate (IP_3_). IP_3_ stimulates the release of Ca^2+^ from the endoplasmic reticulum. There are two major steps leading to the activation. First, mutagenesis and single-molecule studies show that Ca^2+^ binds to C2 domain and mediates the engagement of PKC on the plasma membrane through bridging the C2 domain to phosphatidylserine and PI(4,5)P_2_ (Nalefski and Newton, 2001; Corbalán-García et al., 2003; Evans et al., 2006). Thus, the C2 domain is removed from the kinase domain and the regulatory and catalytic domain are separated. Second, PKC binds its membrane-embedded activator DAG via the C1 domain (Giorgione et al., 2003; Dries et al., 2007; Ziemba et al., 2014), which results in an allosteric conformational change that releases the PS from the substrate-binding cavity and the full activation of PKC (Orr and Newtont, 1994). It should be noted that the number of C1 domains involved in the membrane binding is different among PKC isoenzymes (Ziemba et al., 2014; Antal et al., 2015).

Studies of energetics suggest that membrane binding of C1 and C2 provides the energy to release the PS domain from the active site (Newton and Johnson, 1998). However, the atomic-level structure of the full-length active/inactive PKC and the spatiotemporal activation sequence has remained elusive. The details of how the intra-molecular rearrangement enables the PS release are still unknown. The nature of all domain-domain interactions also remains to be understood.

## 4 C2 domain-containing enzymes

C2 domains have a common fold of an antiparallel β-sheet sandwich (Sutton et al., 1995). The majority of C2 domains interact with lipids in a Ca^2+^-dependent manner. Some C2 domains bind to anionic lipids like phosphatidylserine and phosphoinositides (PIPs) and some bind to zwitterionic lipids like PC (Nalefski et al., 1998; Perisic et al., 1998; Verdaguer et al., 1999; Sánchez-Bautista et al., 2006).

### 4.1 Phosphatase and tensin homolog

Phosphatase and tensin homolog (PTEN) desphosphorylates PI(3,4,5)P_3_ to PI(4,5)P_2_ in the PTEN/PI3Kα/Akt signaling pathway. The product PI(4,5)P_2_, also the allosteric activator, facilitates PTEN hydrolysis of PI(3,4,5)P_3_, which creates a positive feedback loop (Campbell et al., 2003). The catalytic activity of PTEN is stimulated by 5–8 fold in the presence of vesicles containing PI(4,5)P_2_ (McConnachie et al., 2003; Walker et al., 2004). Molecular modeling and NMR studies identified the allosteric site, the N-terminal PI(4,5)P_2_ binding domain (PBD), near the active site (Wei et al., 2015). Mutagenesis studies have shown that PTEN binds to the plasma membrane with three major anchor points (Walker et al., 2004; Shenoy et al., 2012; Wei et al., 2015; Masson et al., 2016; Irvine et al., 2019; Jang et al., 2021): the PBD, the arginine loop in the catalytic phosphatase domain, and the lysine-rich, phosphatidylserine-binding motif CBR3 loop in the Ca^2+^-independent C2 domain.

**FIGURE 5:**
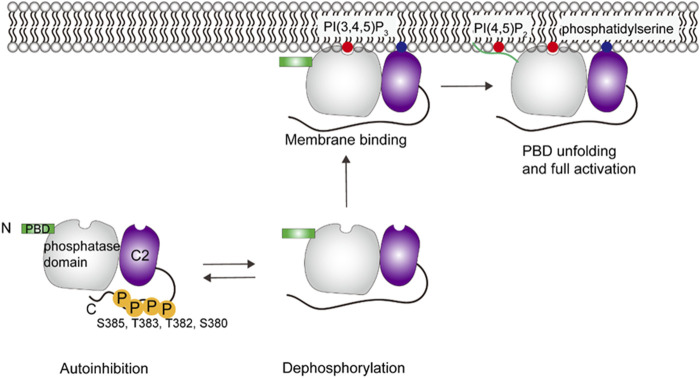
Schematic illustration of PTEN activation. PTEN remains in an autoinhibited state until removal of phosphate groups in the C-terminal region, which then allows membrane interaction. Specific interactions include C2 domain interacting with phosphatidylserine and the PBD domain interacting with PI(4,5)P_2_, which then allows the phosphatase domain to gain access to its PI(3,4,5)P_3_ substrate. This figure was adapted in part from ref. Jang et al. (2021).

X-ray scattering, HDX-MS and cross-linking studies show that the phosphorylated C-terminal tail (CTT) (S380, T382, T383, S385) covers the CBR3 loop and Cα2 in C2 domain and the phosphatase domain, which interferes with the membrane-binding interface and results in a closed, inactive state of PTEN in the cytosol (see Figure 5) (Odriozola et al., 2007; Rahdar et al., 2009; Bolduc et al., 2013; Chen et al., 2016; Masson et al., 2016). PBD binds to the phosphatase domain at the same time. Dephosphorylation of the CTT and the following release of autoinhibition expose the active site (Bolduc et al., 2013). PTEN then binds to the membrane using the arginine loop and CBR3 loop. MD simulations suggest that the coordination of PI(3,4,5)P_3_ to the P loop, one of the core loops of the active site, may allosterically promote unfolding of the α-helix PBD (Jang et al., 2021). The unfolded PBD is subsequently released from the phosphatase domain in the presence of PI(4,5)P_2_, translocated onto the membrane, which stabilizes the protein through salt bridges between lipids and a polybasic patch of the PBD, leading to the coordination of two PI(4,5)P_2_ and full activation of PTEN (Jang et al., 2021).

There are some questions about the PTEN regulation that remain to be studied. First, since CTT is an intrinsically disordered region (IDR), it is difficult to obtain a picture of the proposed closed state (Maccario et al., 2007; Malaney et al., 2013; Dempsey et al., 2021; Smith et al., 2022). Second, previous MD simulations showed that membrane binding of PTEN leads to an orientation change between the phosphatase and C2 domain (Kalli et al., 2014), which requires experimental evidence. In addition, it has been proposed that the full activation of PTEN requires the formation of PTEN dimer (Papa et al., 2014; Heinrich et al., 2015). PTEN dimers seem to have a more compact conformation, which allows for better cooperativity between the phosphatase and C2 domains that may increase of the catalytic activity (Heinrich et al., 2015). How PTEN dimers engage in the PTEN-membrane interaction remains to be understood.

### 4.2 Src homology 2 domain-containing inositol 5-phosphatase

The Src homology 2 domain-containing inositol 5-phosphatase (SHIP) dephosphorylates PI(3,4,5)P_3_ to generate PI(3,4)P_2_. The SHIP family, consisting of SHIP1 and SHIP2, share a high level of sequence and structural homology, but have different tissue expression patterns. A previous study (Ong et al., 2007) showed that adding PI(3,4)P_2_ to the enzyme reaction activates full-length SHIP1, but not SHIP1 without the C2 domain. Protein lipid overlay (PLO) assays suggested that purified C2 domain bound to PI(3,4)P_2_, although there are some concerns about PLO assays being artifact prone. These data indicate that the C2 domain is needed for the allosteric activation of SHIP1 and that allosteric activators such as PI(3,4)P_2_ might bind to the C2 domain.

A recent study (Waddell et al., 2023) used single molecule total internal reflection fluorescence (TIRF) microscopy to visualize membrane association and dissociation dynamics of fluorescently labeled SHIP1 on supported lipid bilayers (SLBs). They found that the SHIP1 truncation containing the central phosphatase domain flanked by C2 domain binds to PI(3,4)P_2_ and pleckstrin homology-related (PHR) domain (PHR-PP-C2) binds to PI(3,4,5)P_3_. Molecular dissection showed that the full-length SHIP1 was autoinhibited by the N-terminal Src homology 2 (SH2) domain and the disordered C-terminus compared to the central PHR-PP-C2. They also found that phosphatidylserine enhanced the activity of full-length SHIP1, but the overall activity was lower than that of PHR-PP-C2 lacking autoinhibition. They suggest that the autoinhibition of SHIP1 is regulated by a mechanism that is partially resistant to phosphatidylserine-mediated activation, and phosphatidylserine may bind to the C2 domain, leading to allosteric activation.

### 4.3 SHIP2

Studies by the Lietha lab established that the C2 domain stabilizes the phosphatase (Ptase) domain and promotes membrane binding (Le Coq et al., 2017). With surface plasmon resonance (SPR) experiments, they found that both the Ptase and Ptase-C2 bind phosphatidylserine, and the C2 domain enhances the binding. The C2 domain affects the catalytic activity of the Ptase domain, which may indicate allosteric communication. MD simulations and X-ray crystal structure analysis suggested that communication between the C2 domain and the active site in the Ptase is modulated by the conformational dynamics of α5–7 helices and L4 loop**.**

More recent MD simulations (John et al., 2023) with SHIP2 bound to a lipid membrane suggest that C2 causes conformational changes in L4 and α5–7, but there is no change in flexibility in these regions, which may be because that the presence of a lipid membrane changes protein conformational dynamics. Another study (Le Coq et al., 2021) focusing on the function of the PHR domain of SHIP2 showed that the reaction product PI(3,4)P_2_ induces higher activity of PHR-Ptase-C2 compared to Ptase-C2 and Ptase. The direct binding of the PHR domain and PI(3,4)P_2_ was indicated by a fluorescence polarization assay. They proposed a model where both the PHR and C2 domains interacted with the membrane, resulting in optimal positioning toward the substrate and allosteric activation of SHIP2. They also proposed allosteric communication via the PHR-Ptase linker interacting with the Ptase-C2 linker.

A simulation and calculation work also compared the role of C2 domains in PTEN and SHIP2 (John et al., 2023). This study showed that the C2 domain of PTEN plays an important role in membrane binding and adjusts the posture of PTEN for substrate binding which is similar to the role of other C2 domains. The C2 domain in SHIP2 binds relatively weakly to phospholipid membranes but contributes to allosteric interdomain changes essential for catalytic activity.

## 5 PH domain-containing enzymes

Pleckstrin homology (PH) domains consist of a 7-stranded β sandwich structure together with a C-terminal α helix (see Figure 3) (Lemmon et al., 1995). They are found in many types of proteins and are thought to bind PIPs and target proteins to specific membranes. The specific interactions with PIPs are still unclear for most PH domain-containing proteins (Singh et al., 2021).

### 5.1 Protein kinase B

Ser and Thr kinase Akt, also known as protein kinase B (PKB), is one of the key proteins in the phosphoinositide 3-kinase signaling pathways. There are three Akt isoforms, Akt1, Akt2 and Akt3, that all have an N-terminal PH domain, an unstructured linker sequence, a kinase domain, and a C-terminal regulatory domain. PI(3,4,5)P_3_ or PI(3,4)P_2_ binding to the PH domain allosterically activates Akt by unmasking the substrate binding site (Ebner et al., 2017).

**FIGURE 6:**
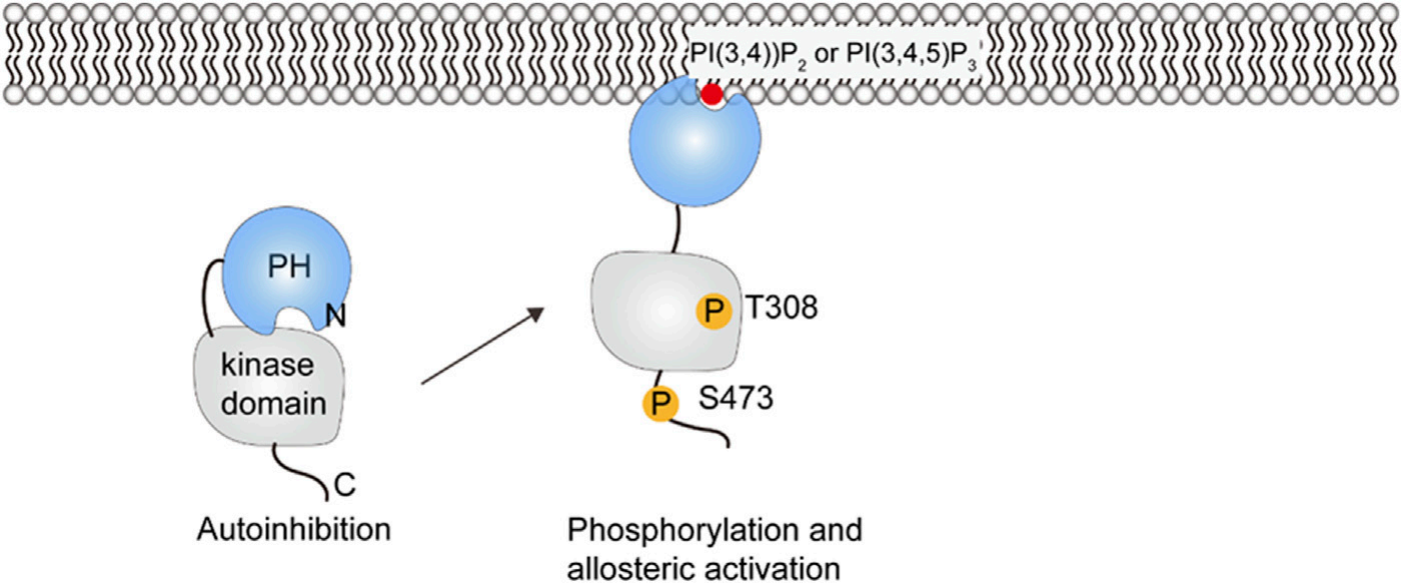
Schematic illustration of PKB/Akt activation. Phosphorylation at positions 308 and 473 releases the PH domain so that it can interact with PIP lipids in the membrane.

The conformational changes of Akt upon PI(3,4,5)P_3_ binding have been elucidated by small-angle X-ray scattering (SAXS) and HDX-MS experiments (Lučic et al., 2018). In the cytosol, the interaction between the PH domain and the kinase domain inhibits substrate binding and keeps Akt in a closed and inactive state, with the activation loop and the hydrophobic motif sequestered in the autoinhibited conformation. The generation of PI(3,4,5)P_3_ or PI(3,4)P_2_ in the plasma membrane leads to the binding of Akt and release of autoinhibition, the PH domain is displaced from the catalytic cleft, the activation loop and the hydrophobic motif are exposed for phosphorylation(see Figure 6) (Ebner et al., 2017). The phosphorylation of T308 by 3-phosphoinositide-dependent kinase 1 (PDK1) orders the activation loop and helps with substrate binding (Stokoe et al., 1977; Alessi et al., 1997), and phosphorylation on S473 by mTOR complex 2 (mTORC2) in the hydrophobic motif orders the C helix and organizes the catalytic residues (Sarbassov et al., 2005; Lučic et al., 2018). The phosphorylated residues are protected by ATP from dephosphorylation (Yang et al., 2002a; Yang et al., 2002b). Fluorescence imaging techniques and live cell spectroscopy show that dissociation from PI(3,4,5)P_3_ is the rate-limiting step in Akt dephosphorylation and the presence of PI(3,4,5)P_3_ is required for sustained Akt phosphorylation (Ebner et al., 2017). Turnover of PI(3,4,5)P_3_ and PI(3,4)P_2_ results in Akt inactivation by returning it to the autoinhibited form. The phosphorylated activation loop and hydrophobic motif are released and then accessible for dephosphorylation (Lučic et al., 2018). In summary, both PI(3,4,5)P_3_/PI(3,4)P_2_ binding and phosphorylation are required for Akt activation. The significance of activation in the presence of PI(3,4,5)P_3_ or PI(3,4)P_2_ is thought to enhance the substrate specificity and reduce the potential crosstalk between signaling pathways (Ebner et al., 2017).

### 5.2 Bruton’s tyrosine kinase

Bruton’s tyrosine kinase (Btk) is a protein kinase that is critical in activation of B cells. Btk consists of a PH domain fused with a Tec homology (PH-TH) domain, a proline-rich region (PRR), a Src homology 3 (SH3) domain, a SH2 domain, and a C-terminal kinase domain. The activation of Btk depends on the recruitment to the plasma membrane. The PH-TH domain binds two PI(3, 4, 5)P_3_, which allosterically mediate Btk dimer formation and activation (Chung et al., 2019; Wang et al., 2019).

**FIGURE 7:**
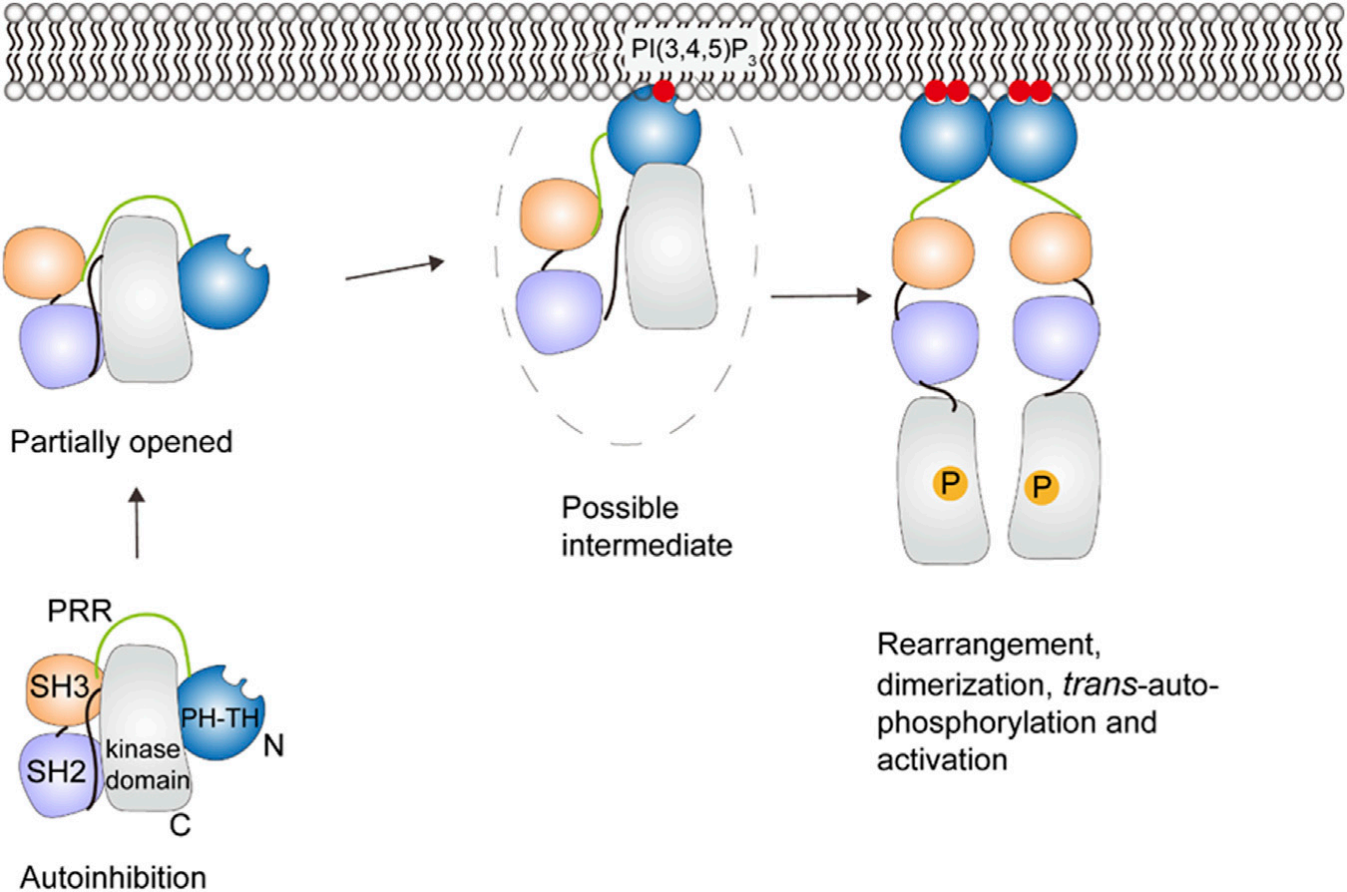
Schematic illustration of Btk activation. A series of conformational changes, including changes in domain-domain interactions, must occur to allow the PH-TH domain to interact with PI(3,4,5)P_3_. There is a proposed intermediate state that interacts with the membrane, but further conformational changes, dimerization and *trans*-autophosphorphorylation are required for full activation. This figure was adapted in part from ref. Kueffer et al. (2021).

NMR and HDX-MS reveal the autoinhibitory interactions in cytosolic Btk between the PH-TH and the kinase domain, and between SH3 and the SH2-kinase linker (see Figure 7) (Devkota et al., 2017; Joseph et al., 2017; Amatya et al., 2019). Displacement of the SH3 domain from the kinase domain by PRR transiently opens the autoinhibited structure of Btk (Joseph et al., 2017), which may promote PI(3, 4, 5)P_3_ binding. After Btk is recruited to the membrane containing PI(3, 4, 5)P_3_, a domain rearrangement can occur and likely leads to a second autoinhibitory structure with the PH-TH domain adopting the Saraste dimer (Hyvönen and Saraste, 1997), a structure associated with membrane binding. The second autoinhibitory structure based on crystallography (Wang et al., 2015)may suggest an intermediate between the fully autoinhibited and active state (Amatya et al., 2019; Kueffer et al., 2021). When there is sufficient amount of PI(3, 4, 5)P_3_ in the membrane, Btk binds to PI(3, 4, 5)P_3_ through both the canonical and peripheral sites on the PH domain to stabilize membrane interactions (Chung et al., 2019). The binding of the second PI(3, 4, 5)P_3_ may also be involved in an allosteric structural change or electrostatic interaction necessary for dimerization (Chung et al., 2019; Wang et al., 2019). This further structural change allows the SH2 domain to contact the kinase domain N-lobe and helps stabilize the kinase domain in its active state. Btk then undergoes *trans*-autophosphorylation and achieves activation (Rawlings et al., 1996).

Fluorescence correlation spectroscopy (FCS) shows that by binding to multiple PI(3, 4, 5)P_3_, the PH-TH module is more sensitive to the PI(3, 4, 5)P_3_ concentration on the surface thus leading to better regulation of Btk (Chung et al., 2019). However, the role and structural basis for the PI(3, 4, 5)P_3_-induced dimerization in Btk activation is not clear and awaits further study.

### 5.3 Myotubularins

Myotubularins (MTM) are phosphatidylinositol phosphatases, which dephosphorylate the D-3 position of PI3P and PI(3,5)P_2_ and generate phosphatidylinositol and PI5P, respectively (Begley and Dixon, 2005). The product PI5P is an allosteric activator that promotes oligomerization of MTM1 into a heptamer, which enhances the activity of MTM1 (Schaletzky et al., 2003). The PH domain overlapping with the Glucosyl transferases, Rab-like GTPase activators and myotubularins (GRAM) motif has been reported to bind PIPs and mediate the allosteric activation (Lorenzo et al., 2005). The structural details about the activation and the oligomerization require further studies.

### 5.4 Phospholipase D

Phospholipase D (PLD) is a transphosphatidylase that hydrolyzes PC to generate phosphatidic acid (PA) (Mohn et al., 1992). Two canonical mammalian isoforms PLD1 and PLD2 have similar structures (Sung et al., 1999a; Sung et al., 1999b). The tandem phox homology-pleckstrin homology (PX-PH) domains in PLDs are thought to activate the enzyme by association with PI(4,5)P_2_, and they may play different roles in membrane associations and activation in different PLD isoforms (Yao et al., 2021). A recent study based on crystallography and mutagenesis demonstrated that a polybasic pocket in the catalytic domain likely binds PI(4,5)P_2_ (Bowling et al., 2020). Structural determination of full-length PLD is also needed to identify the PIP binding sites and activation mechanisms.

## 6 PX domain-containing enzymes

PX domains are membrane binding domains that bind to phosphatidylinositol and PIPs and consist of three anti-parallel β-strands followed by three α-helices (see Figure 3) (Ellson et al., 2002). Besides PLD, PX domain also exists in serum- and glucocorticoid-regulated kinase 3 (Sgk3). Sgk3 is a serine/threonine protein kinase allosterically activated by PI3P. The allosteric activation process of Sgk3 is similar to that of Akt. HDX-MS provides evidence for conformational changes in Sgk3 in the presence of vesicles (Pokorny et al., 2021). Cytosolic Sgk3 is autoinhibited by its PX domain, PI3P binding relieves the autoinhibition and renders Sgk3 a substrate for PDK1. Then PDK1 phosphorylates the Sgk3 activation loop, which completes the activation of Sgk3 (Kobayashi and Cohen, 1999).

## 7 FERM domain-containing enzymes

Four-point-one, ezrin, radixin, moesin (FERM) domains have three compact lobes (see Figure 3) and are found in a variety of cytoskeletal-associated proteins that link the plasma membrane with cytoskeleton at specific cellular locations (Chishti et al., 1998; Frame et al., 2010).

### 7.1 Focal adhesion kinase

Focal adhesion kinase (FAK) is a non-receptor tyrosine kinase and is composed of a N-terminal FERM domain, a central kinase domain, an unstructured proline-rich region, and a C-terminal focal adhesion targeting (FAT) domain. FAK binding to PI(4,5)P_2_-rich membranes in focal adhesion induces a multistep activation sequence, which causes FAK oligomerization and an conformational change for autophosphorylation and activation (Goñi et al., 2014; Acebrón et al., 2020).

**FIGURE 8:**
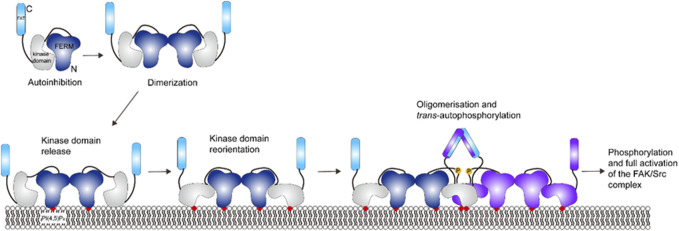
Schematic illustration of FAK activation. Activation of FAK requires dimerization to release the kinase domain and allow for domain reorientation. FAK can further oligomerize upon *trans*-phosphorylation. This figure was adapted in part from ref. Acebrón et al. (2020).

In the autoinhibited form, the FERM domain binds to the kinase domain, blocks the active site and sequesters phosphorylation sites (see Figure 8) (Lietha et al., 2007). FAT domain targets FAK into focal adhesions (Arold et al., 2002; Hayashi et al., 2002; Gao et al., 2004). Locally increased concentration of FAK close to the adhesome induces FAK dimerization through FERM-FERM interactions (Brami-Cherrier et al., 2014). Vesicle pull-down experiments show that a basic patch on the FERM domain binds to PI(4,5)P_2_ in the membrane through electrostatic interactions (Goñi et al., 2014). Cryo-electron microscopy (cryo-EM) of FAK bound to a PI(4,5)P_2_ -containing membrane shows that binding induces FAK oligomerization between FAK dimers and release of the autoinhibition: the kinase domain is released, and it undergoes a conformational change, binds to PI(4,5)P_2_ in the membrane and places the active site towards the membrane (Hall and Schaller, 2017; Acebrón et al., 2020). The conformational change upon PI(4,5)P_2_ binding also exposes Y397 in the linker between the FERM and kinase domains to *trans* -autophosphorylation by another FAK dimer. The Src kinase is then recruited to the autophosphorylated linker and phosphorylates Y576 and Y577 in the activation loop of FAK, which activates FAK (Calalb et al., 1995; Lietha et al., 2007). The active site of FAK faces toward the membrane in the cryo-EM structure, making it difficult for Src to access the activation loop and for substrates to access FAK catalytic site. An additional level of regulation, likely the tension force, is thought to expose the active site and trigger the activation of FAK in the presence of PI(4,5)P_2_ (Acebrón et al., 2020). The understanding of tension force within the focal adhesion requires further experiments. In addition, the proximity of FAK may promote FAT dimerization (Le Coq et al., 2022) through a helix-swapped structure observed in crystallography (Arold et al., 2002), which has not been proven since FAT domain is missing from the cryo-EM structure (Goñi et al., 2014).

## 8 Other enzymes regulated by membrane-interactions

There are other enzymes that are allosterically regulated by membranes, but most mechanisms are unclear. PI(4,5)P_2_ binds to a basic region of regulatory domain in P21 activated kinase 1 (Pak1) to promote its activation synergistically with Rac1/Cdc42 (Strochlic et al., 2010; Malecka et al., 2013). Arachidonic acid was shown to activate protein phosphatase 5 (PP5) by binding to its regulatory tetratricopeptide repeat (TPR) domain (Chen and Cohen, 1997). Another example is Sac1, where its activity is stimulated by binding of anionic phospholipids like phosphatidylinositol and phosphatidylserine. Lipid binding in the cationic groove induces a conformational switch of the catalytic P-loop and activates Sac1 (Zhong et al., 2012).

## 9 Discussion

Here, we have described the mechanism of membrane binding and subsequent conformational changes for key peripheral membrane binding enzymes. These actions not only re-localize these enzymes to these membranes, but also modulate their catalytic activity. We have categorized enzymes into where an amphipathic α -helix or a specialized lipid-binding domain is most important for membrane engagement. The amphipathic α-helices in CCT and PLA_2_ bind to the core of the protein in the unbound, inactive form. Rotation of these helices exposes the hydrophobic side for binding to the membrane. Amphipathic helices enable the protein to sense changes in physical properties of the bilayer. Thus, proteins containing amphipathic helices not only bind to specific lipids but respond more generally to negative surface charge and lipid packing voids of the membrane.

In contrast, lipid-binding domains consist of a binding pocket for a specific lipid, especially different phosphoinositide lipids, and enable the protein to be regulated by the concentration of a specific lipid in the membrane. In addition, the presence of more than one lipid-binding domain allows cooperation between lipids to increase the binding affinity and enzyme activity, diversifying enzyme regulation. For example, regulation of PKC’s function by two lipid-binding domains provides higher sensitivity in the activation process.

The general regulation strategy used by many peripheral proteins is to displace an autoinhibitory domain or release the intramolecular interaction upon membrane binding. Some peripheral proteins undergo oligomerization or dimer dissociation during membrane binding, where the changes of intramolecular and intermolecular interactions both contribute to the allosteric regulation. Moreover, positive feedback via allosteric activation by reaction products is observed in some phosphoinositide phosphatases, which is an important part in lipid metabolism. These processes are also often induced by changes in the phosphorylation state of these proteins. Other post-translational modifications (PTMs), especially palmitoylation and acylation, are also important for membrane recruitment and are responsible for fine-tuning enzyme function. Cysteine palmitoylation attaches a long chain fatty acid to the protein, increases its hydrophobicity and membrane-binding affinity (Salaun et al., 2010). Palmitoylation regulates function and cellular localization of Btk-C isoform and Akt. The phosphorylation levels of Btk-C and Akt are decreased if they are unpalmitoylated (Blaustein et al., 2021; Kokabee et al., 2022). Palmitoylation of PKC facilitates its interaction with membrane (Ford et al., 1998). Lysine acetylation neutralizes the positive charge of the protein, resulting in weaker binding with negatively charged membranes (Okada et al., 2021). Acylation at the PH domain of Akt blocks membrane-binding and reduces Akt phosphorylation, while deacylation promotes membrane-binding and activation (Sundaresan et al., 2011). Acylation of PTEN in the catalytic cleft attenuates its activity (Okumura et al., 2006).

While much has been revealed about membrane binding and consequent allosteric regulation, high-resolution protein-membrane binding structures from X-ray crystallography or cryo-EM are still required to map the spatiotemporal activation sequence for most enzymes mentioned above. Imaging studies to reveal the co-localization of membrane-bound proteins with specific lipids *in vivo* may also help us to understand how membrane localization and allosteric activation happen inside cells. These studies will continue to reveal important concepts in membrane binding and allosteric regulation, and provide novel mechanisms by which to modulate these functions through small molecules, serving both as chemical probes and potential starting points for pharmaceutical development.
